# Study of the Digital Teaching Competence of Physical Education Teachers in Primary Schools in One Region of Spain

**DOI:** 10.3390/ijerph17238822

**Published:** 2020-11-27

**Authors:** Jorge Rojo-Ramos, Jorge Carlos-Vivas, Fernando Manzano-Redondo, María Rosa Fernández-Sánchez, Jara Rodilla-Rojo, Miguel Ángel García-Gordillo, José Carmelo Adsuar

**Affiliations:** 1 Motricity and Education (HEME) Research Group, Department of Health, Economy, University of Extremadura, 10003 Cáceres, Spain; jorgerr@unex.es (J.R.-R.); jorge.carlosvivas@gmail.com (J.C.-V.); safriblue@hotmail.com (J.R.-R.); jadssal@unex.es (J.C.A.); 2 Department of Education Sciences, Faculty of Teacher Training, University of Extremadura, 10003 Cáceres, Spain; rofersan@unex.es; 3 Faculty of Administration and Business, Autonomous University of Chile, Sede Talca 3467987, Chile; miguelgarciagordillo@gmail.com

**Keywords:** information technology and communication, primary school teacher, physical education, professor, digital competence

## Abstract

In today’s society and, in the teaching profession especially, it is demanded that we have a remarkable digital competence and have continuous formation in technological recycling. This study intends to describe and expose the levels of digital competency amongst physical education teachers working in the public school system of Spain, by what was established in the portfolio of teacher digital competence of one region of Spain which was published on the 12th of June 2015 in the Official Journal of Extremadura. The design of our research is of a descriptive type. The instrument used to collect data was the questionnaire published in appendix IV of the previously mentioned portfolio official of a region of Spain. A total of 201 students were tested. The principle obtained results show that primary physical education teachers in public schools in Spain have a basic level of digital teaching competence, more specifically an A2 level. This is in comparison with the guidelines established by the Common Framework for Digital Competence of Teachers 2.0.

## 1. Introduction

We are in a society where new digital technologies are part of our daily lives. As citizens, we are required to be digitally competent to actively participate in this Digital Society [1]. Even so, it is more important and relevant as teachers, where it is demanded that we have a remarkable digital competence and have a continuous formation in technological recycling. This process of continuous updating [1], recycling, and the acquisition and development of new competencies and/or skills, depending on the requirements of the former, is very important in the educational field, which is constantly advancing.

It is undeniable that digital technologies are very present in our lives. According to Rodríguez et al. [2], the educational context is one of those which has developed the most, and specifically in the teaching and learning processes, making new didactic models and active methodologies possible. Related to, Marqués [3] states about Information and Communication Technologies (ICT) that “when we join these three words, we are referring to the set of technological advances provided by computers, telecommunications, and audiovisual technologies...These technologies provide us with information, tools for their process, and communication channels” (p. 3).

To refer to digital competence and, in relation to the educational field, we will focus on the definition of the European recommendation of 2018 [4]: “Digital competence implies the safe, critical and responsible use of digital technologies for learning, at work and for participation in society, as well as interaction with these technologies. It includes information and data literacy, communication and collaboration, media literacy, digital content creation (including programming), security (including digital well-being and cyber-security related skills), intellectual property issues, problem- solving and critical thinking” (p. 9).

In this sense, Ortega-Sánchez et al. [5] define the teacher’s digital competence as the set of knowledge, skills, and attitudes needed to be functional in a digital teaching environment and a correct process of teaching and learning.

As far as the inclusion of ICT in the current education system is concerned, the road traveled to reach the present moment is relatively short; the first appearance in the curriculum was in the Organic Law on the General Organisation of the Spanish Education System (1990) [6]. Later, in the Organic Law on the Quality of Education [7], the references to be implicitly called Information and Communication Technologies disappeared, forming an element of modernity that must be implemented in the classroom. At present, the education system is regulated by the Organic Law for the Improvement of Educational Quality [8] and includes ICT from the stage of childhood education throughout the education system.

By the above, digital technologies have brought about a pedagogical revolution that, with their correct use, could lead to deeper and more significant learning, favor technological experiences [9], and notably more dynamic learning [10].

The inclusion of ICT in education should be understood as an interactive process between subjects, tools, and contexts, and therefore they should not be considered as isolated elements. Colás and Jiménez [11] explain that the incorporation of ICTs into classroom educational contexts requires teachers to achieve skills of an instrumental, systematic, and applied nature.

Physical education is a curricular area of Primary Education. In principle, we could think that there is no relationship between physical education and ICT. However, Obrador and Campos-Rius [12], state that there are some common practices of technologies that manage to improve the motivation, effectiveness, and efficiency of the teaching of physical education.

Following Berrocoso et al. [13], the Educational Technology Network constitutes the incorporation of the one region of Spain Educational System into the Information Society, which includes both infrastructures and the creation of a space where research, formation, and innovation in the field of ICT can be promoted in this region.

Berrocoso and Diaz [14], however, state that the process of integration of ICTs is made up of different levels of implementation and that the summit would be the process of transformation and real integration of ICTs. These levels are mainly three: introduction or assimilation, application or transition, and integration or transformation. The level of introduction or assimilation corresponds to the provision and generalized access to technological resources to the school. That is, what we could call a first step towards the digital literacy of the educational community. The level of application or transition would correspond to the second stage. The teachers already have specific training, and have acquired a series of basic skills, and are preparing to discover web resources, to optimize the assimilation of the content provided or arranged from other, in this case, more traditional methods. Finally, the integration or transformation process takes place at the moment in which the technological resources are applied and used in the educational center as a tool for access and production of knowledge, as well as for group collaboration and communication elements. Responsible attitudes towards the use of new technologies must be generated and the autonomy of students must be promoted through the acquisition of digital competencies [14].

According to Níkleva and López Ogáyar [15], “digital competence means analyzing information critically through personal, autonomous, and cooperative work. It is also about knowing how to use technological resources to solve real problems efficiently” (p. 126).

On the one hand, the Ministry of Education and Science, in Royal Decree 1631/2006, of 29 December [16], defines Digital Competence as “having the skills to search for, obtain, process and communicate information and to transform it into knowledge” (p. 688).

On the other hand, the National Institute of Educational Technologies and Teacher Formation specifies that Digital Competence is one of the eight fundamental competencies that a student must have developed adequately once they have finished their stage of compulsory education, to be able to adapt to adult life positively.

The current legislative frameworks in the field of education, being these the Organic Law 2/2006, of 3rd May on Education [17], the Organic Law 8/2013, of 9th December for the improvement of educational quality [8], and the Law 4/2011, of 7th March, on Education of Extremadura [18], refers to the integration of information and communication technologies in the educational work and the administrative management of the centers.

The National Institute of Educational Technologies, through the publication of the Common Framework for Digital Teaching Competence, to regularise digital competences and based on the Digital Competence Framework for Citizens (DIGCOMP) 2.0 project [19], has established five areas to group together each of the digital competencies that a teacher must possess which refer to information and information literacy; communication and collaboration; creation of digital content; digital security and problem-solving [20].

For the elaboration of this research study, these five dimensions or areas of digital competence defined by the National Institute of Educational Technologies and Teacher Formation [20] and the Portfolio of Digital Teaching Competence of one region of Spain that primary education teachers must possess have been taken as a reference. Each area has a series of competencies related to it, always taking the Common Framework of Digital Teaching Competence as a reference. Previously, as a starting point, we must refer to the Education and Formation 2020 Strategic Framework (ET2020), which aims to serve as an updated strategic framework for European cooperation in the field of education and formation based on the achievements of its predecessor (ET2010).

In addition, among the conclusions reached by the Council [4], four main strategic objectives were set for the above framework: making lifelong learning and mobility a reality; improving the quality and effectiveness of education and formation; promoting equity, social cohesion, and active citizenship; and enhancing creativity and innovation, including entrepreneurship, at all levels of education and formation.

The initial version of the Common Framework for Digital Teaching Competence, on which the Digital Teaching Competence Portfolio of one region of Spain is based, lays the foundations for the DIGCOMP project [19], which was started in 2010 by the European Commission’s Institute for Prospective Technological Studies (ITPS) and whose author is Anusca Ferrari.

Ferrari [15], in the DIGCOMP 1.0 project [19], established five areas of competence, as detailed above, but also developed a series of competencies that integrated each area.

**Figure 1:**
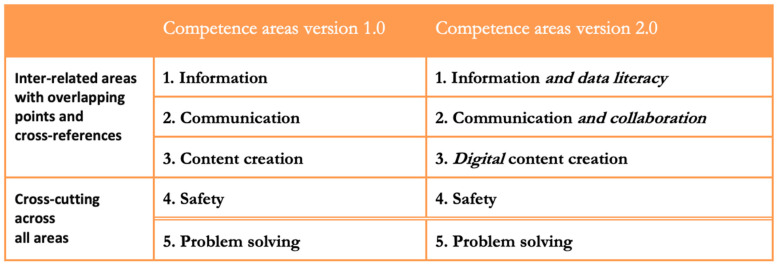
Competence areas version 1.0 and 2.0.

In the DIGCOMP 2.0 project [19], published in 2016, some additions (Figure 1) were made to the descriptors established in version 1.0 as well as to the competencies assigned to each of the five areas. Below is a figure comparing the areas established in both versions.

Once these modifications were incorporated into the areas of competence (dimension 1), changes were also made in dimension 2, establishing new additions to the competencies established for each of the areas. These are the following:

1. Information and data literacy.
  1.1. Browsing, searching, and filtering data, information, and digital content.
  1.2. Evaluation of data, information, and digital content.
  1.3. Management of data, information, and digital content
2. Communication and collaboration.
  2.1. Interaction through digital technologies.
  2.2. Sharing through digital technologies.
  2.3. Participation in citizenship through digital technologies.
  2.4. Collaboration through digital technologies.
  2.5. Netiquette.
  2.6. Digital identity management.
3. Creation of digital content.
  3.1. Integration and reworking of digital content.
  3.2. Copyright and licenses.
  3.3. Programming.
4. Digital Security.
  4.1. Protection of devices.
  4.2. Protection of personal data and privacy.
  4.3. Protection of health and welfare.
  4.4. Protection of the environment.
5. Problem-solving.
  5.1. Resolution of technical problems.
  5.2. Identification of needs and technological responses.
  5.3. Using digital technologies creatively.
  5.4. Identification of digital competence gaps.

Therefore, the General Secretariat of Education, in its resolution of 2 June 2015, published in the DOE on Friday, 12 June 2015, based on version 1.0 of the DIGCOMP project [19], published the Portfolio of Digital Teaching Competence of one region of Spain.

Thereby, in the first instance, a document that guides teachers in evaluating their situation compared to the established standards or areas, and in the second instance, to guide their formation needs so that they are adjusted to their demands and needs.

Within the established areas, the different descriptors are distinguished, to materialize an assessment of them, defining six levels, grouped into three blocks: Beginner User—A1 and A2; Intermediate User—B1 and B2; Advanced and Expert User—C1 and C2.

In line with the above, Blanco and Amigo [21], state that digital technologies are revolutionizing all elements of the educational context, but that there is a need to adapt educational methodologies and paradigms to suit the needs of students in this technological age. These authors [21] state that teachers are challenged to acquire digital knowledge, skills, and attitudes that motivate students to make critical use of technology, both in the educational sphere and in their social life and leisure environments, building a collective and exciting response to the challenges posed to education today by the Digital Age. As a teacher, it is no longer only important to acquire digital skills, but also to adapt your full role to complete effective teaching.

Some of these practices which the authors point out are based on the fact that since the majority of students have access to mobile devices with an internet connection, educational practices can be carried out in open spaces outside the traditional physical education classroom. Another of the utilities that make technology and physical education converge are blogs and audiovisual files such as Youtube. After reviewing the digital tools used in the physical education classroom and which allow us to improve these processes, we will proceed to present the most significant ones: Classdojo, the Google Sites tool, tools such as Kahoot or Socrative, Geocaching, virtual learning platforms such as Moodle, etc.

In summary, some authors such as Obrador and Campus-Rius [12] show us, there are a large number of ICT tools that support the teaching of physical education and that provide teaching processes with added value, as they normally simplify the management of certain teaching practices, allow the classroom to be gamma-edged and increase student motivation.

However, despite all the support of the aforementioned authors, works such as Padilla [22] reveal that many teachers do not have acceptable formation to be able to use ICTs efficiently and effectively and also show a lack of attitude and preparation.

As Leiva and Moreno [23] states, the future teachers who are currently in their initial formation have a lack of digital formation, because it may think that the results that expose this lack of preparation and useful use of digital technologies only occur in teachers who are active, but unfortunately this is not the case [23]. The lack of adequate mastery of digital competencies linked to the teaching role coincides with those obtained in the study by Colás-Bravo et al. [24] in the context of secondary education, where it is concluded that “teachers through their educational practice develop the digital competence of their students at an intermediate level” (p. 31).

In this way, the first aim of the research will be to describe the self-perception of Primary School Physical Education teachers on their self-perceived competence and how they consider their digital competence, based on the portfolio of digital teaching competence in one region of Spain. In connection with this, to compare the digital competence of Primary School Physical Education Teachers in public schools in one region of Spain with the levels established in the Common Framework for Digital Teaching Competence. Likewise, the second aim of an attempt will be made to describe whether Physical Education teachers integrate ICT and involve them in the teaching-learning processes in the Physical Education classroom.

## 2. Materials and Methods

Our paper is based on a methodology of descriptive cut through which we want to determine the level of digital teaching competence that a population of physical education teachers in primary schools in one autonomous community of Spain.

The universe (N) is of 419 active teachers in the area of Physical Education in primary schools in one region of Spain. The sample size (*n*) is 201 active teachers of Physical Education in public schools in one Autonomous Community of Spain, for a confidence level (NC) of 95% and a margin of error of ±5%, in the assumption of maximum variability of the proportion (p/q = 50%). The type of sampling has been random since each element of the universe has had the same probability of being selected for the study. As for the distribution of the sample, 79 belong to the province of Badajoz and 122 to the province of Cáceres.

The instrument for collecting the information relating to the object of study in this work consisted of the use of the form published in Annex IV of the Portfolio of Digital Teaching Competence in one region of Spain [18], so no validation was required, as this is a document published by the General Secretariat of Education, which is intended to serve as a tool for assessing the Digital Competence of one region of Spain teaching staff. We transferred the questionnaire to the Google Forms tool so that it could be filled in digitally and the results recorded in real-time. The e-form is made up of 239 items grouped into each of the five areas of competence, in addition to a first block designed to collect information to identify and profile the teacher. The justification for the choice of this instrument (e-formulary) is mainly based on this instrument allows greater savings in costs and time and is quite precise. It also offers improved presentation, a higher rate of return, and assured and rapid delivery.

## 3. Results

The technique used for the analysis of the data obtained was quantitative in the case of the questionnaire applied to Physical Education teachers in public primary schools in one region of Spain.

In this section, we will present the results obtained in the research divided into two sections. The first one shows the results obtained regarding the demographic data of the sample participants. The second section corresponds to the analysis of the results obtained from the answers provided by the teachers in each of the items corresponding to the application of the e-questionnaire published in the portfolio on digital teaching competence to a population of physical education teachers in primary schools in an Autonomous Community of Spain.

### 3.1. Demographic Data

#### 3.1.1. Sex

To carry out the research, we carried out a questionnaire using Google forms, from which we obtained the participation of 201 people. Of these, 54.2% (*n* = 109) are men and 45.8% (*n* = 92) are women.

#### 3.1.2. Age

**Figure 2:**
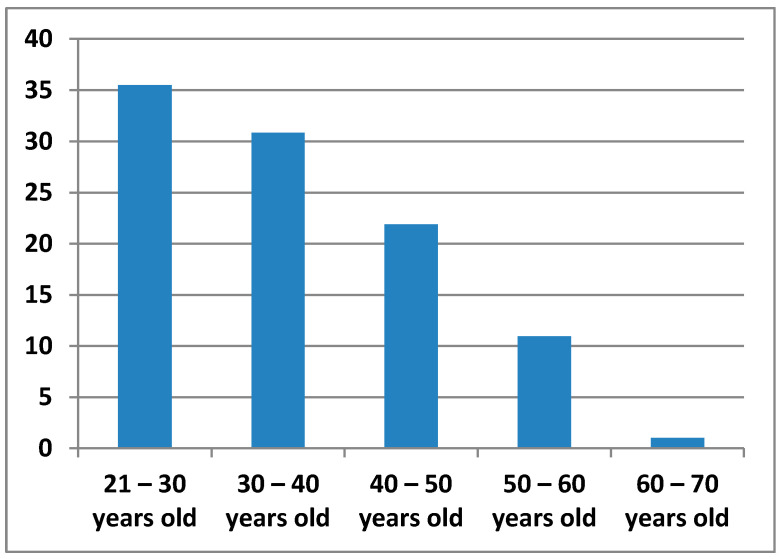
Percentage of age groups.

The age ranges proposed for obtaining data on teachers are 21 to 30 years old; 30 to 40 years old; 40 to 50 years old; 50 to 60 years old; and 60 to 70 years old (Figure 2).

#### 3.1.3. Province

**Figure 3:**
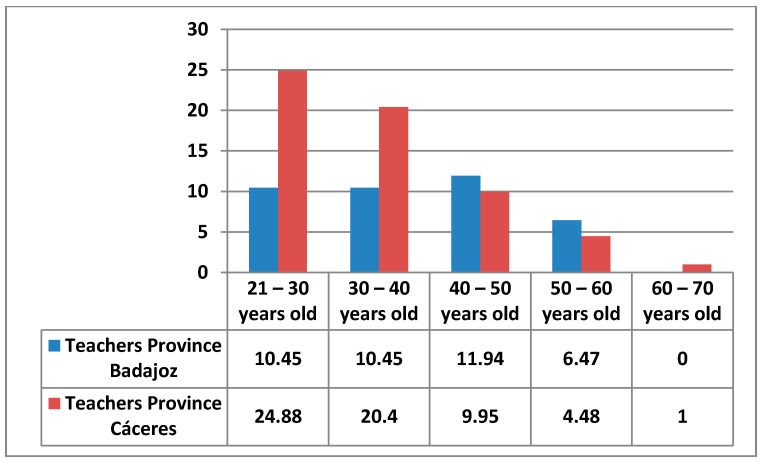
Percentages of age and province.

The teachers surveyed come from one region of Spain, with 39.3% from the province of Badajoz and 60.7% from the province of Cáceres (Figure 3).

#### 3.1.4. Centre’s Educational Environment

**Figure 4:**
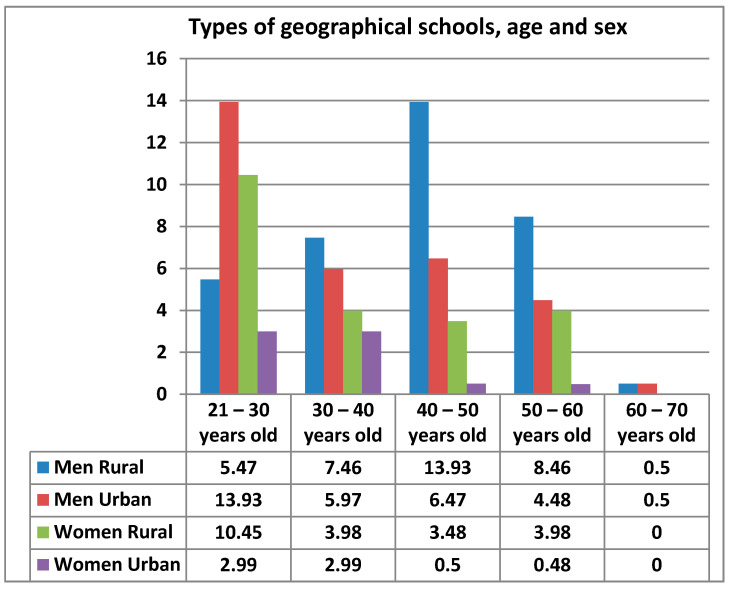
Relationship by types of geographical schools, age and sex.

There are only two types of geographical areas where schools are located: urban and rural. The percentage of teachers teaching physical education in urban schools is 42.29% and in rural schools is 57.71%. The percentages between the types of school and the different age ranges among the teachers surveyed can be seen below (Figure 4).

#### 3.1.5. Teaching Posts

**Figure 5:**
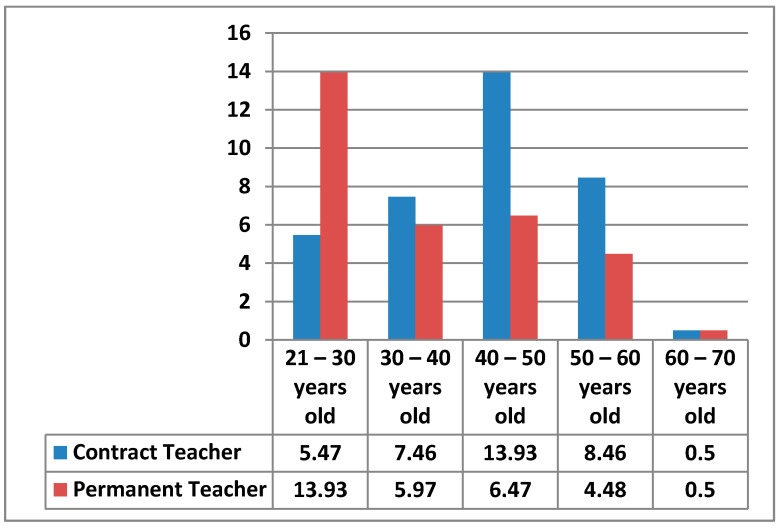
Teaching posts by age.

The types of posts occupied by the teachers surveyed were contract or permanent posts, with 45.77% contract teacher positions and 54.23% permanent teacher positions. In the following figure (Figure 5), we can see how these percentages are divided by type of post and age.

Contract teacher positions between 21–30 years old stand out in the graph with about 30.35%, followed by those with a permanent teacher position between 40–50 years old with 19.40%. In third place were those with permanent teacher positions between 30–40 years old (18.41%), followed by the same range of 30–40 years old for contract teacher position. The age ranges with the lowest percentage were people with a permanent teacher position in the 50–60 age range (10.45%), people with a permanent teacher position between 21–30 years old (4.98%), contract teacher position of 40–50 years old (2.49%), people with a permanent teacher position of 60–70 years old (1%) and, finally, contract teacher position between 50–60 years old (0.5%).

#### 3.1.6. Teaching Experience

**Figure 6:**
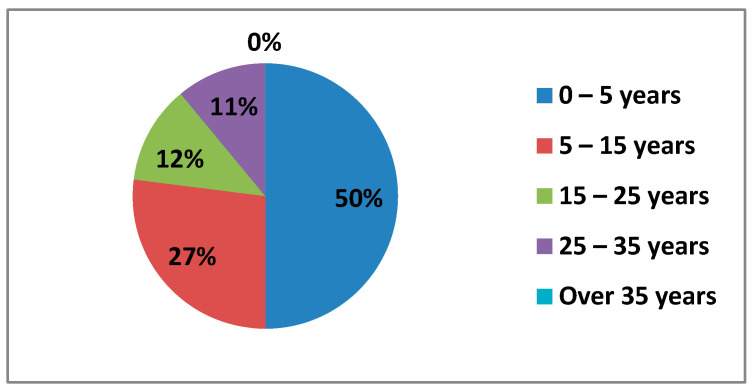
Teaching experience.

The data obtained about the experience of the teachers surveyed were in the following ranges of teaching experience: 0–5 years; 5–15 years; 15–25 years; 25–35 years; and over 35 years (Figure 6).

As we can see, 50% of the teachers surveyed had a teaching experience of 0–5 years, followed by teachers with 5–15 years’ experience with 27%. The ranges of teaching experience between 15–25 years (12%) and 25–35 years (11%) were more equal and the percentage of teachers with more than 35 years’ experience was 0.5%.

#### 3.1.7. Level of Self-Perceived Digital Competence

**Figure 7:**
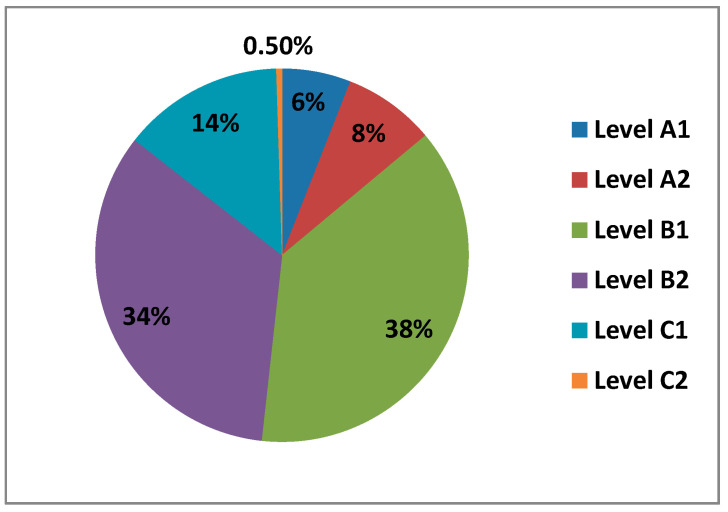
Teachers by level of self-perceived digital competence.

The questionnaire asked teachers what level of digital competence they thought they had overall in all areas. In the following figure (Figure 7) we can see the different levels.

We observe how most teachers perceive having a B1 level (38%), followed by a B2 level (34%) and a C1 level (14%). Levels A2 (8%), level A1 (6%) and level C2 (0.5%), are the levels with the lowest percentages.

#### 3.1.8. Specific ICT Formation after Initial Teacher Formation

**Figure 8:**
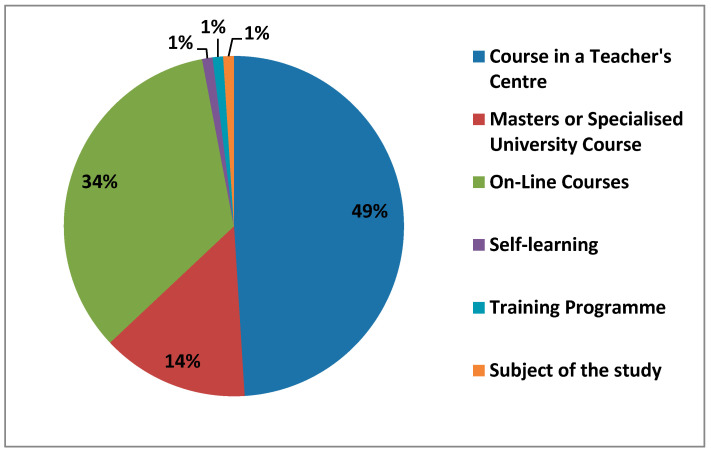
Post-study ICT formation for teachers.

Teachers have been trained in ICT in various ways. The options offered in the questionnaire were Course in a Teachers’ Centre, Master’s or Specialised University Course, Online courses on distance learning platforms, or the option of ‘Others’ where teachers could write down which option they had taken to improve their ICT formation. In the figure (Figure 8) below we show the percentage that each of them obtained.

The option of complimentary formation with Course in a Teachers Centre had the highest percentage (49%), followed by online courses on distance learning platforms (34%) and university masters or specialized courses (14%). The answer with the option to be filled in by the teachers themselves as Other, in which a total of 3.5% of the answers were obtained, divided into Self-study (1%), the formation program of the Diputación de Cáceres (1%), the degree course (1%) and the ICT course (0.5%).

### 3.2. Level of Digital Competence

**Figure 9:**
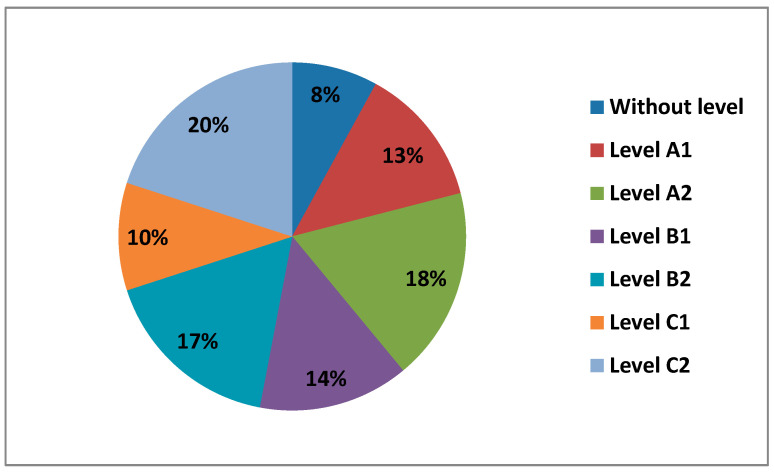
Average levels of digital teaching competence.

Digital competence was measured in five different areas: Area 1—information; Area 2—communication; Area 3—content creation; Area 4—digital security; and Area 5—problem resolution. Each area had six different levels, which correspond to a more global level: Level A1 and Level A2 (Beginner User Level), Level B1 and Level B2 (Intermediate User Level), and Level C1 and Level C2 (Advanced User Level). In the following figure (Figure 9) we can see the average percentage that each level had in all the areas.

As we can see, teachers averaged a higher percentage at Level C2 (20%), followed by Level A2 (18%), then Level B2 (17%), Level B1 (14%), Level A1 (13%) and Level C1 (10%). We can also see how an average number of teachers had no level at all in digital competence (8%).

#### 3.2.1. Area 1—Information

**Figure 10:**
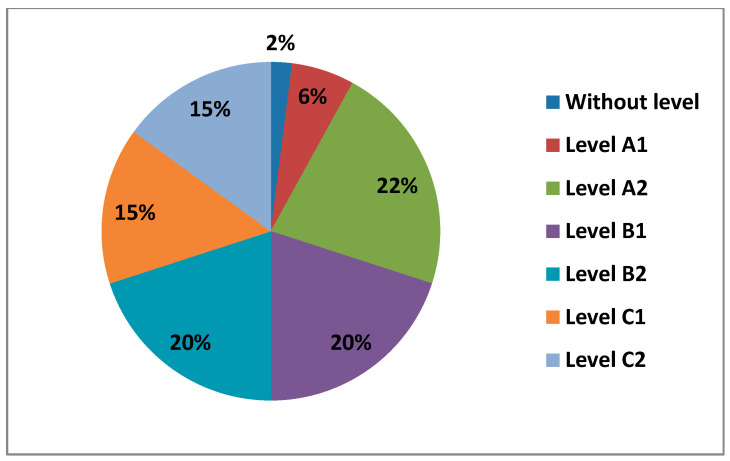
Percentage of teachers by level of digital competence in Area 1.

Next, we are going to see the data obtained from Area 1—information, in its different levels, which are obtained by fulfilling the items corresponding to each level (Figure 10).

As we can see from the graph above, Level A2 (22%) was the level where most teachers were found in this area. It was followed by Levels B1 and B2 with the same percentage (20%) and Levels C1 and C2, also with the same percentage (15%). Finally, we found Level A1 (6%) and a small percentage of teachers who did not have any level in this area.

**Figure 11:**
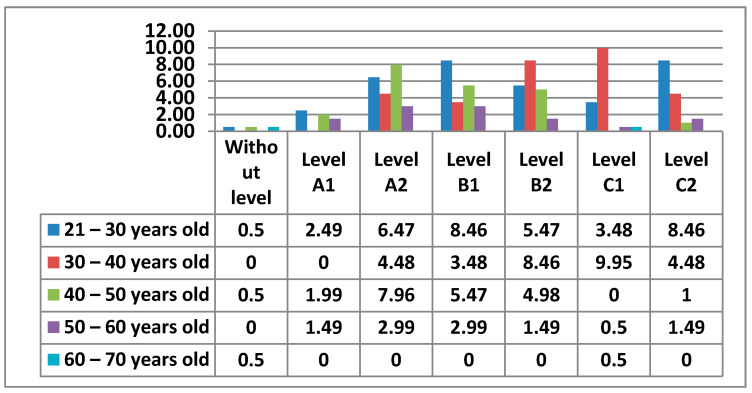
Level of digital competence in teachers by age in area 1.

The following figure (Figure 11) shows the Level of digital competence that teachers had in Area 1—information, according to their age.

**Figure 12:**
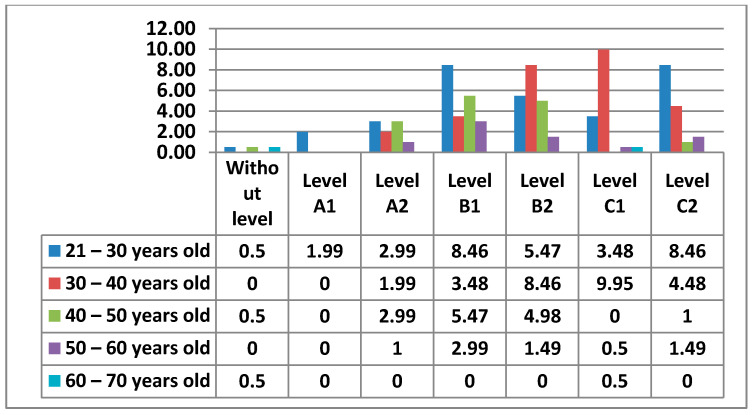
Level of digital competence in women by age in area 1.

**Figure 13:**
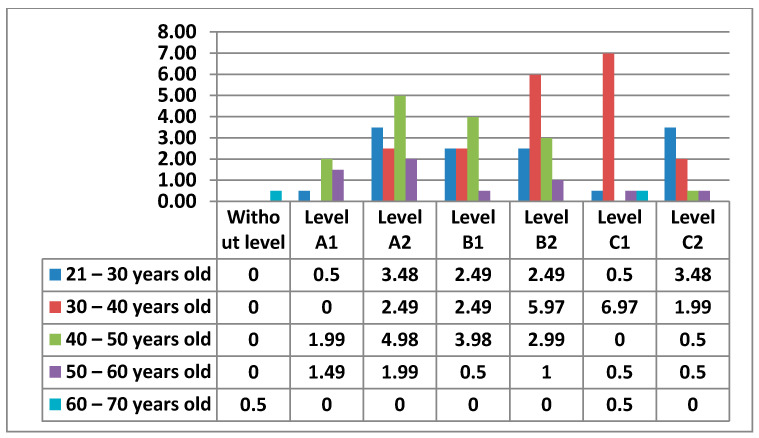
Level of digital competence in men by age in area 1.

Below, we can see the graphs showing the percentages of digital competence level by sex and age in Area 1, which we will compare according to the most outstanding data (Figure 12 and Figure 13).

#### 3.2.2. Area 2—Communication

**Figure 14:**
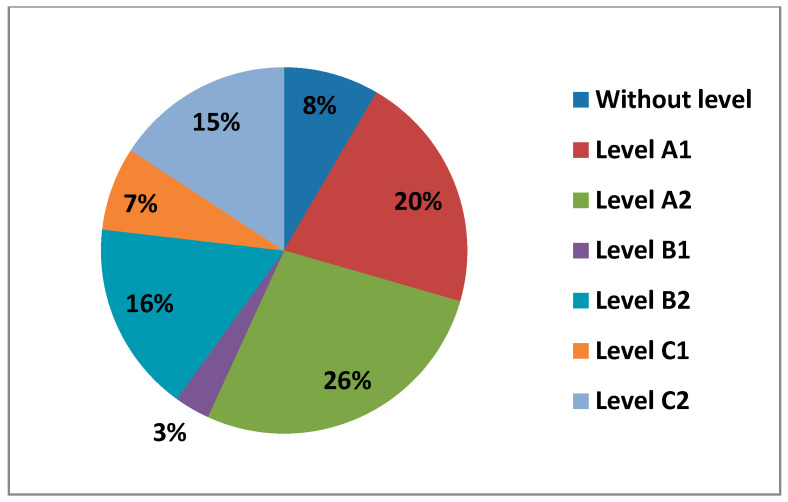
Level of digital competence in teachers in Area 2.

In this Area 2—communication, teachers with Level A2 (26%) stood out, followed by those with Level A1 and Level C1 (20%), then we found those with Level B2 (16%) in fourth place. In fifth place, and with the lowest percentages, we found teachers with a C1 level (7%) and those with a B1 level (3%) in the last place. It is quite visible that there was a percentage in this Area that did not have any level (8%) (Figure 14).

**Figure 15:**
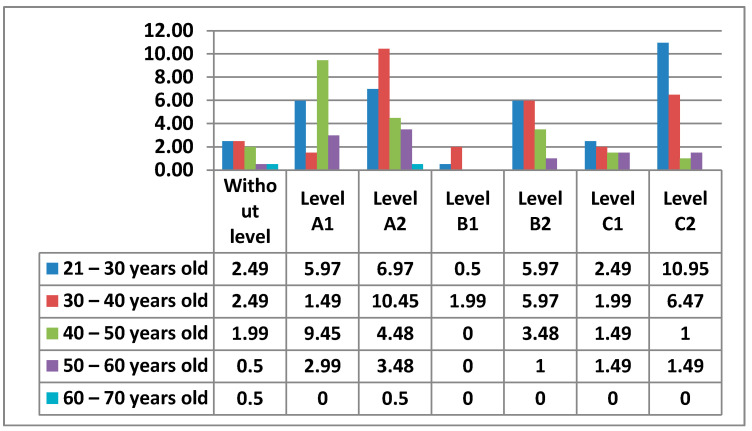
Level of digital competence in teachers by age in area 2.

The figure below (Figure 15) shows the percentages of the levels that teachers had by age.

**Figure 16:**
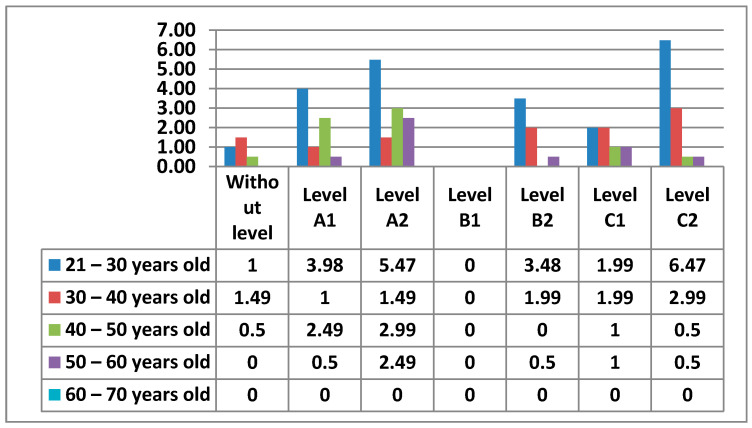
Percentage of women’s digital competence by age in area 2.

**Figure 17:**
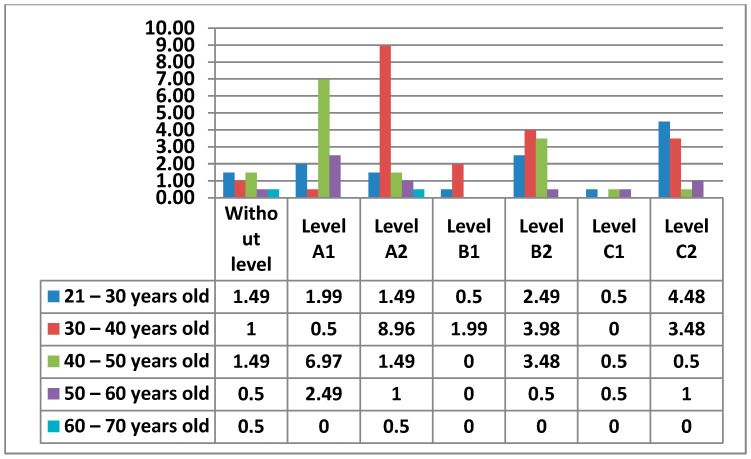
Percentage of men digital competence by age in Area 2.

In the following figures (Figure 16 and Figure 17) we see the levels of teachers compared by sex and age in this Area 2—communication.

#### 3.2.3. Area 3—Creation of Digital Content

In Area 3—content creation, the percentages of the levels that teachers had were more equal.

**Figure 18:**
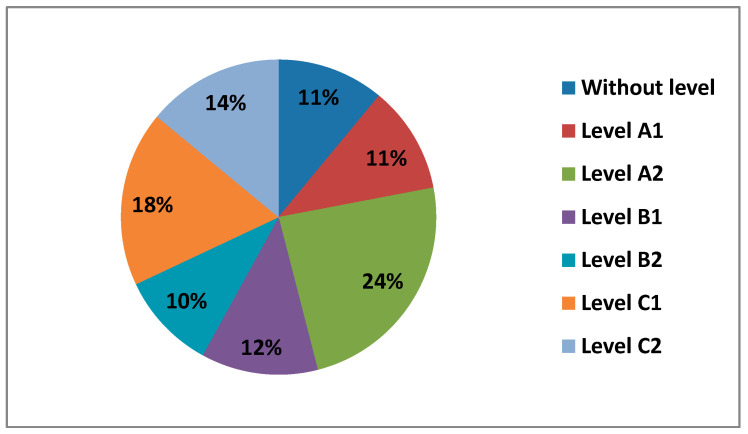
Level of digital competence in teachers in area 3.

The following figure (Figure 18) shows the different percentages of the levels that teachers had in Area 3—content creation.

Firstly, we see that Level A2 stood out the most (24%), followed by Level C1 (18%), Level C2 (14%), Level B1 (12%), Level A1 (11%), and Level B2 (10%).

We also see that there were 11% of teachers who did not have any level in Area 3—content creation.

**Figure 19:**
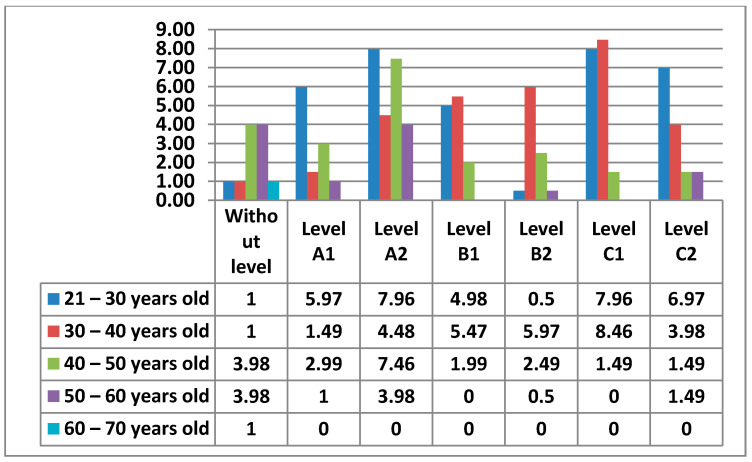
Digital competence in teachers by age in Area 3.

Next, we will see in the Figure 19 the data obtained from all the levels by age.

**Figure 20:**
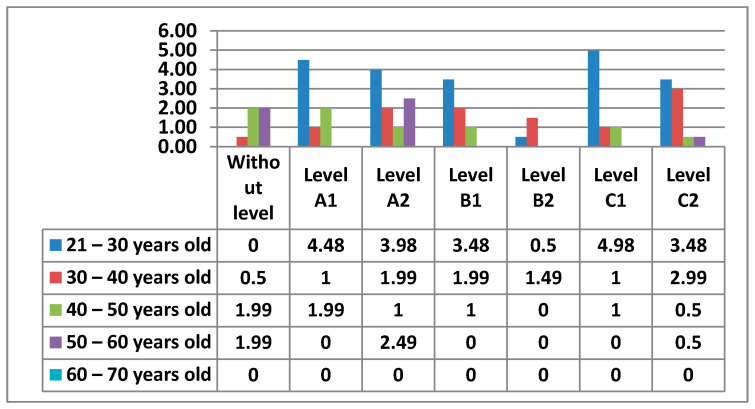
Digital competence in women by age in Area 3.

**Figure 21:**
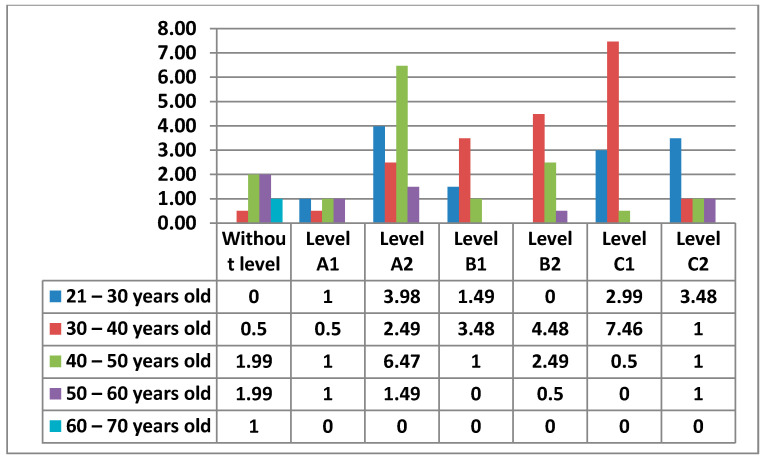
Digital competence in men by age in Area 3.

Next, we will look at the data obtained from the levels in this Area 3—content creation, by women (Figure 20) and by men (Figure 21).

#### 3.2.4. Area 4—Digital Security

**Figure 22:**
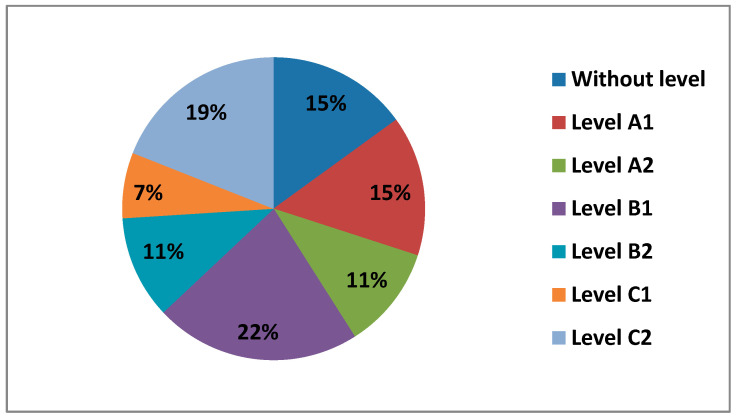
Level of digital competence in teachers in Area 4.

The following figure (Figure 22) shows the percentage of teachers at different levels in Area 4—Safety.

**Figure 23:**
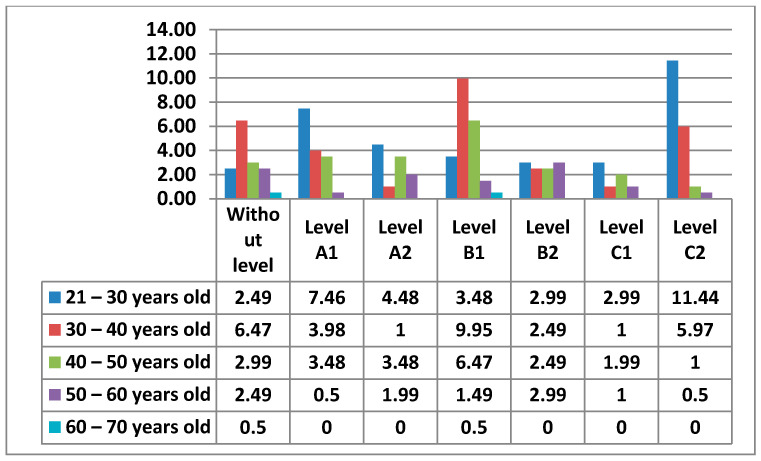
Digital competence in teachers by age in Area 4.

In the following figure (Figure 23) we can see the different percentages obtained by level and age in this Area 4—digital security.

**Figure 24:**
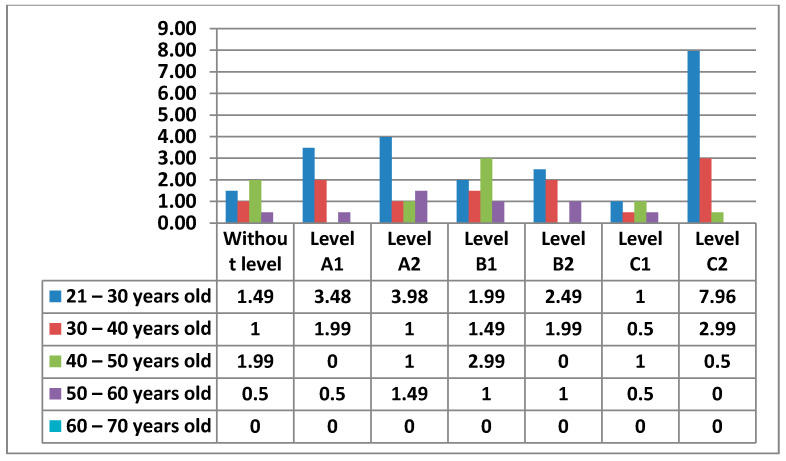
Digital competence in women by age in Area 4.

**Figure 25:**
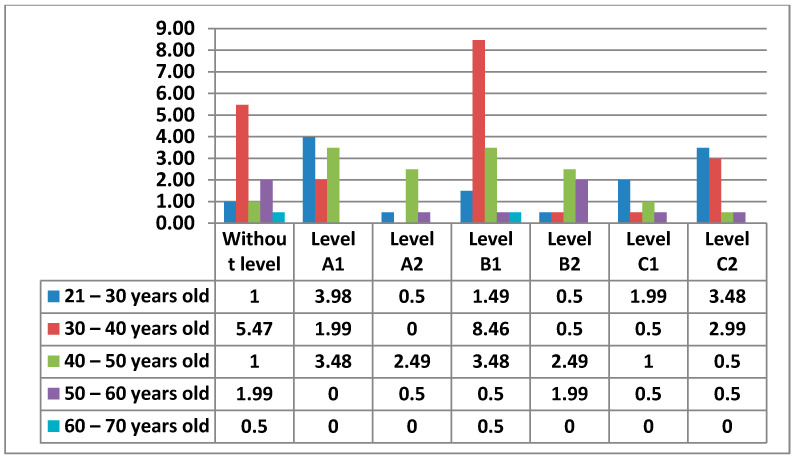
Digital competence in men by age in Area 4.

The figures below contain the data obtained from the percentages of women (Figure 24) and men (Figure 25) by level and age.

#### 3.2.5. Area 5—Problem Solving

**Figure 26:**
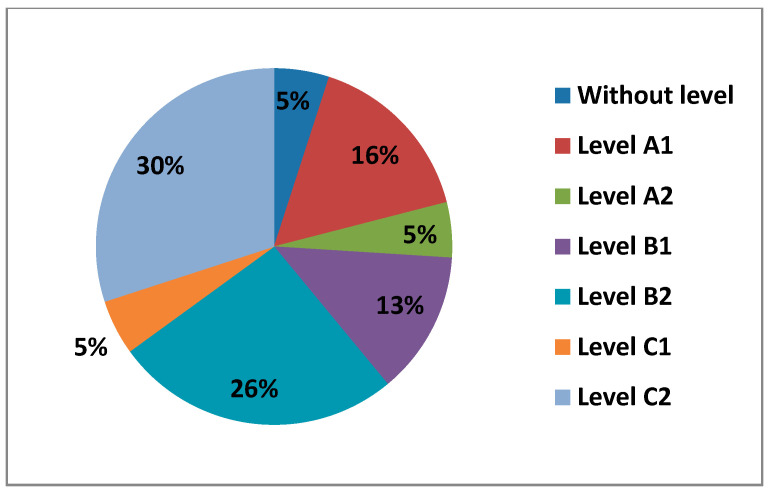
Level of digital competence in teachers in Area 5.

In the following figure (Figure 26) is the percentage according to the levels that teachers had in Area 5—problem solving.

**Figure 27:**
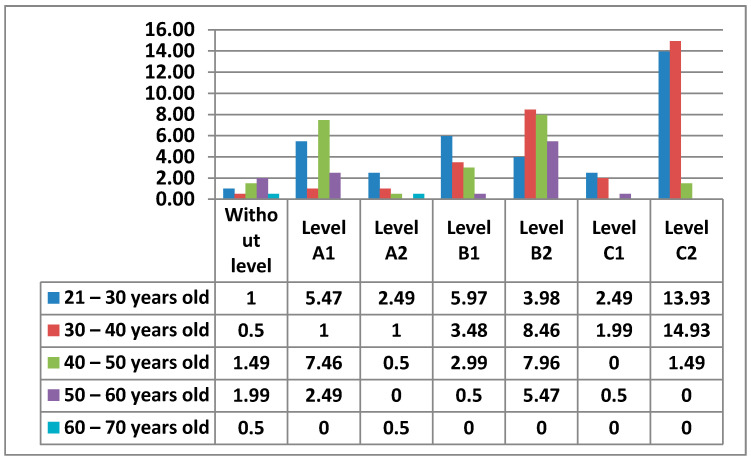
Digital competence in teachers by age in Area 5.

The following figure (Figure 27) shows in more detail the different levels of teachers by age.

**Figure 28:**
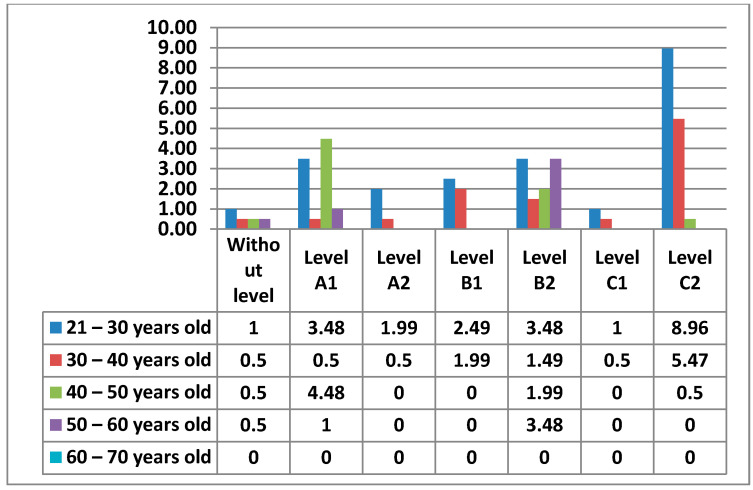
Digital competence in women by age in Area 5.

**Figure 29:**
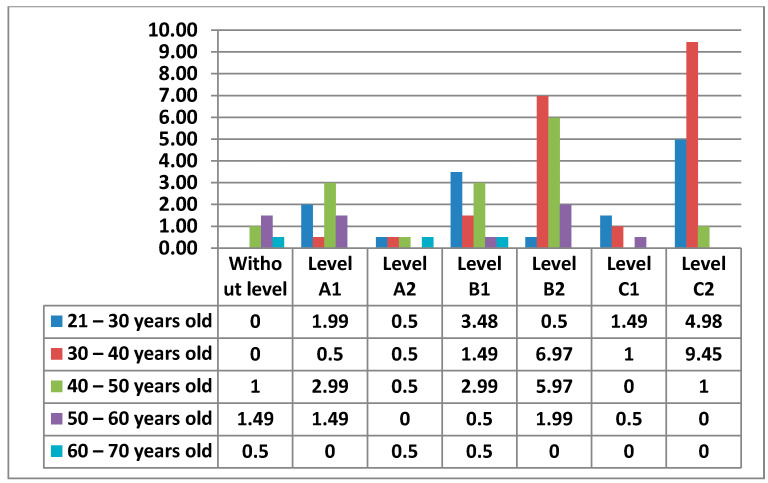
Digital competence in men by age in Area 5.

In the following figures (Figure 28 and Figure 29) we have broken down the percentages we have found in women and men by level and age.

## 4. Discussion

Digital competence is considered a fundamental skill in teachers, a basic requirement to favor the integration of ICT in the classroom and improve teaching and learning processes, as some authors point out [25,26,27]. Poor self-perceived competence is the impediment observed in some researches [28,29] that can inhibit the use of educational technology in the classroom, as teachers are the decisive agent for its integration [30,31].

Next, we will discuss the results we have obtained in this research which has sought to authenticate the questionnaire on digital competence and that we know as such the level of this competence. The sample of the study is composed of 201 physical education teachers (109 men and 92 women) from childhood and Primary Education School in one region of Spain. Therefore, 54.23% corresponding to men and 45.77% corresponding to women.

Once the results are known, we can say that the difference between men and women participating in the questionnaire is not very significant, being at 8.4%. The percentage of women who have participated in the questionnaire between 21–30 years old is considerably higher than that of men in this same age group. However, there is greater participation by men in the 30–40 age groups, as well as in the 40–50 age groups. The percentage of participants is equal in the 50–60 age brackets but stands out the participation of men in the 60–70 age brackets, as women in this age bracket have not participated.

In the case of the province that has obtained the most participants, it has been the province of Cáceres, with almost 20% difference. If we look at these data compared by sex, we find that the majority of participants in the questionnaire have been women between 21–30 years old and men between 21–40 years old, both as we have already mentioned, work in centers in the province of Cáceres.

Based on the environment of the center, with a 15% difference, we found that more participants are working in rural schools than in urban schools.

About the types of positions occupied by the teachers surveyed, we found differences between contract teacher position and permanent teacher positions. When comparing the types of positions held by teachers, we found that there are more female contract teacher positions than male contract teacher position aged 21–30 and that there is almost twice as much difference between male contract teacher position and female contract teacher position in the 30–50 age range.

The percentage of teaching experience that stands out the most is that of 0–5 years, which is half of the people surveyed. This is because most of the people who have participated in the survey are those between 21–30 years old, who have, for the most part, a contract teacher position. The second most striking percentage is that of the 5–15-year age range, which also coincides with the percentage of people working with a permanent teacher position in the 30–50 year age group.

There is a significant difference between levels B1 and B2, which indicate an intermediate level of digital competence. This may be since we already live in the information age, where ICT is already quite integrated into our lives, both professionally and personally. The levels of self-perceived digital competence, if we compare it by sex, can hardly be observed differences in their percentages. The only level we see that is different, where women have no presence, is at level C2. When we see the percentages of the different levels of self-perceived digital competence compared by age, we observe that the great difference is found in levels B1 and B2 between 21–40 years old, decreasing little by little between 40–60 years old. When teachers are asked about the level of digital teaching competence they think they have, they mostly answer that a B1 and B2, that is, in other words, that the average level they perceive they have on digital competence is intermediate, followed by a basic level, where we find level A1 and level A2 and, finally, they perceive they have an advanced level, level C1, and level C2. The C1 level is dominated by men between 30–40 years old, with their percentage being almost 5 times higher than that of women at this same level and age group. Concerning C2, as we have already mentioned, despite being a rather low percentage, it is made up of men between 21–30 years old.

It can be seen how teachers take advantage of complementary ICT formation offered by Teachers’ Centres, as these are specific courses for teachers. They are followed by online courses on distance learning platforms as the second most popular option. We have also observed to a lesser extent, how the self-learning method has emerged.

Further ICT formation clearly shows how each age group of teachers prefers one type of formation over another. For example, when we talk about the age group of teachers between 21–30 years old, we have seen that the possibility of carrying out such formation on distance learning platforms where they offer online courses was highlighted. On the other hand, we see how teachers between the ages of 30–50 prefer the formation offered by courses in a teachers’ center, whose courses are generally conducted in a face-to-face mode. We can also see how teachers between 21–40 years old prefer to take a master’s degree or specialized university courses, although to a lesser extent than the results mentioned above. Women have a considerably lower percentage of formation received in ICT through masters or specialized university courses than men. What does show a difference, although not very notable, is that women between 21–30 years old are those who show self-learning of complementary formation.

In the following sections, separated by the different areas, is where we have been able to find out the real level of digital competence that teachers have.

We have seen that the average percentages of the levels in the different areas have been quite equal; however, it should be shown that the average level that teachers have in Physical Activity Education is a Basic Level with the difference of only 1%, followed by an Intermediate Level and an Advanced Level, and differentiated by only 0.6%.

Furthermore, it should be added that there are 8% of teachers who do not have any digital teaching competence.

To measure the level of digital competence of the teachers surveyed, we have carried out different sections in the questionnaire with the corresponding questions for each level and area.

Starting with Area 1—information, the percentages in this Area are quite equal for Level A2, Level B1, Level B2, Level C1, and Level C2. The level with the lowest percentage is Level A1. As we have seen, there are 2% of teachers who have no demonstrable level in this area, as they do not meet any of the minimum requirements for accreditation. Although the highest percentage is at Level C1 in the 30–40 age group, the average level of teachers in Area 1—Information is, without doubt, an Intermediate User Level, as it is levels B1 and B2 that stand out most in all age groups. The percentages found in the data for women in this area are considerably lower than those found in the data for men. Even the levels in which the highest percentages of women stand out are different from those of men. We have observed that in total there is a percentage of teachers who do not have any level in Area 1—information. In the case of women, the age groups that do not have any level in this area are 21–30 years old and 40–50 years old, and in the case of men, it is in the 60–70 years old group. It must be said that the percentage of teachers who have no level in this area is almost nil. In Area 1—information, men aged 30–40 with a C1 level stand out in the first place. On the other hand, the section that stands out among women is 21–30 years old with a B1 level. This same group of women is the second-highest percentage, but with a C2 level. The percentage of men in the 30–40 age group with a B2 level is quite different (almost double) to that of women. We can also see how the 60–70 age group in men has a C2 level in this area.

Continuing with Area 2—communication, the percentages we found were more varied than in Area 1. In Area 2—communication, the teachers with an A2 level stand out. Unlike Area 1—information, in Area 2—communication, we have observed that the percentage of teachers who do not have any level increases considerably. This may be since it is already an area that is more dedicated to the use of new tools, offered by education centers, (Rayuela for public education centers) and their correct use in terms of communication. It is important to highlight that many of the teachers who have been implemented the use of these platforms have not had the necessary formation to use them (or such formation has been very basic). In addition to education platforms, Area 2—communication reflects the use made by teachers of synchronous and asynchronous communication tools, such as social networks for education, a field that is still being studied and applied in many schools in different countries. Among men, we see that in Area 2—communication, the percentage that stands out the most is that of Level A2, with the age group of 30–40 years old (8.96%). It is followed by Level A1 with the age range of 40–50 (6.97%). In the results, we have seen that the highest level that men with 30–40 years old have in Area 2—communication is a Level A2. Women have their highest percentage at Level C2, in the 21–30 age group. The 40–50 age group for men with a Level A1 is the next highest. The second most prominent level for women is Level A2 in the 21–30 age group. Among men we have seen that in all age groups there are teachers who do not have any level. The only age group that coincides in that both men and women have the same percentage of teachers without any level in Area 2—communication, is the 30–40, age group. The average level that teachers have is a Level A2.

In Area 3—content creation, it is identified whether the teachers know how to use different computer programs, on-line programs, or applications, whether they are free or private software, which allows the creation, manipulation, and distribution of information. As in Area 2—communication, in Area 3—content creation, there is a higher percentage of teachers who do not have any level in this area, as it is a question of using tools to create different types of content and even advanced tools for the manipulation or creation of teaching content. There are teachers without any level in all age groups, however, we observe that teachers between 21–40 years old have a lower percentage than those between 40–60 years old. This means although it is not such a high percentage, that despite being born into the digital age, there is still a lack of digital literacy even among the youngest teachers. The percentages of levels are more equal than in previous areas. In Area 3—content creation, the 30–40 and 21–30-year age groups with Level C1 and the 21–30 year age group with Level A2 stand out. The male and female teachers who do not have any level are practically identical. The difference exists between the 21–30 age group, where men have no teacher with any level and women have one, and the 60–70 age group, where the opposite is true. Likewise, while the highest percentage of men in Area 3—content creation is in the 30–40 age group with Level C1, women share the highest percentage between two levels, Level C1 and Level A1, both with the same age group of 21–30 years old.

In Area 4—digital security, we find the percentages of teachers who know or do not know how to protect both their computer equipment and the identities of themselves and their students. This is where digital fingerprinting, digital identity, and the Data Protection Law come into play, knowing their advantages and their limits for the correct use of the contents. In Area 4—digital security, we find that the percentage of teachers who do not have any level in this area is once again increasing. The average level in Area 4—digital security is B1. The percentage that stands out most in Area 4—digital security is Level C2 in the 21–30 year age group followed by Level B1 in the 30–40 year age group. We have seen in the results of Area 4—digital security that there is a higher percentage of men who have no level than women and that the most outstanding age group for men is 30–40 years old, while the 40–50 age group is outstanding for women, although with a percentage four times lower. In terms of the age group that stands out most among men, we are talking about the 30–40 age group with a B1 level, while among women, the most outstanding level is C2 with a 21–30 age group. In both sexes, it can be seen that Level C1, in Area 4—digital security in all age groups, is the one with the least number of teachers.

Finally, in Area 5—problem solving, the knowledge that teachers have to solve the small problems that can appear in the teaching practice with the different electronic devices of the center appears. Unlike the previous areas, this Area 5—problem solving has a higher percentage in one of the most advanced levels, C2. Furthermore, the percentages are higher in the intermediate and advanced levels. It is worth noting that the 21–30 age group has the highest percentage of women who do not have any level in this area, a percentage that does not exist in the data for men. Area 5—problem solving may be the least difficult, since teachers who handle ICT for personal use, which are the vast majority, have the facility to apply such knowledge to the proper functioning of electronic equipment that is in the center. The percentage of men who do not have any level in Area 5—problem solving is quite low, although it is higher than the percentage of women in this same area. Likewise, while the percentage of men is more concentrated in the few age groups that show that they have teachers without any level in Area 5—problem solving, the percentages of women are very well distributed, with almost identical percentages in all age groups. In Area 5—problem solving, it can be seen how both men and women stand out in the same C2 level, differentiated only by the age group that each sex has.

## 5. Conclusions

The results of the questionnaires indicate that we may have a prototype of a male Physical Education Teachers in one region of Spain, who is between 21–30 years old and works in the province of Cáceres, in a rural school and with a permanent teacher position and who has between 0–5 years of teaching experience. This teacher has completed his complementary formation in a Course in a Teachers Centre and has an average level of Basic Teaching Digital Competence.

To be able to affirm the proposals made, we must know whether or not the objectives set are being met. If we now focus on the first objective, concerning to describe the self-perception of Primary School Physical Education teachers on their self-perceived competence, we must say that the teachers answered that they perceived an average Intermediate Level of Digital Teaching Competence, which has been demonstrated with the answers to every one of the items that it is not true. However, and as we said before, they have a Basic User Level.

As for the second objective, concerning to describe whether Physical Education teachers integrate ICT into the teaching-learning processes in the Physical Education classroom, it can be said that the average teacher does not include them. In this case, when we have carried out the analysis by age of the teachers, it can be seen that it is the younger generations who are promoting this use of ICTs in the Physical Education classroom. With all this, it can be confirmed that with the results obtained from the questionnaires carried out on teachers in an Autonomous Community of Spain, they have, although by a small percentage, a Basic User Level of digital teacher competence. This means that, although they have a basic level, they have the basis to continue to advance and achieve teaching digital competence with the complementary formation that is considered appropriate, as this would be the only way for teachers to obtain an average Intermediate or even Advanced Level of digital competence. Despite this, we have been able to see how teachers, who are starting their professional careers as teachers at Physical Education, are increasing the use of ICT in their subject, as far as possible.

Our research has certain limitations, the most notable being the process of evaluation and accessibility to the sample. Regarding the evaluation process, we have to point out that the questionnaire is made up of 239 items, so filling it out turned out to be a dense task and even, according to some of the teachers surveyed, demotivating and frustrating. We have to point out that a significant number of teacher collaborators in the research, showed their complaint towards the instrument through email. The second significant limitation is the accessibility of the sample. Once the schools to which our intervention would be directed were selected, few responded by facilitating contact with the Physical Education specialists, so we had to resort to telephone calls and faxes to insist on their collaboration. As a final limitation, we should also point out a problem that can often encounter in the humanities and social sciences. This is that the data produced was the result of a representation of the respondents on their level of acquisition of digital skills according to the various aspects declined in your reference system and not the result of their actual level.

Based on the data we have obtained, other research can be carried out in the future. One of them will be as analyzing the digital competence of teachers after specific formation for Physical Education teachers and the use of ICT in their subject. We can also compare the data with other primary schools in Spain and even internationally. Further research can be carried out into why future generations of teachers are implementing ICT while more experienced generations are not so much. Furthermore, this data can be compared with data from other existing research in other subjects and educational levels. Therefore, all this research can be developed in the future based on the data we have obtained from this research.
